# Corporal Composition and Gut Microbiome Modification Through Exclusion Dietary Intervention in Adult Patients with Crohn’s Disease: Protocol for a Prospective, Interventional, Controlled, Randomized Clinical Trial

**DOI:** 10.3390/jcm14113998

**Published:** 2025-06-05

**Authors:** Rosario Paloma Cano-Mármol, Virginia Esperanza Fernández-Ruiz, Cristina Martínez-Pascual, Inmaculada Ros-Madrid, Gala Martín-Pozuelo, Alba Oliva-Bolarín, María Antonia Martínez-Sánchez, Juan Egea-Valenzuela, María Ángeles Núñez-Sánchez, Bruno Ramos-Molina, Antonio José Ruiz-Alcaraz, Mercedes Ferrer-Gómez

**Affiliations:** 1 Department of Endocrinology and Nutrition, Virgen de la Arrixaca University Clinical Hospital, 30120 Murcia, Spain; palomacanomarmol96@gmail.com (R.P.C.-M.); virginiaesperanza.fernandez@um.es (V.E.F.-R.); inmarosmadrid@gmail.com (I.R.-M.); galamartin@um.es (G.M.-P.); alba.oliva@imib.es (A.O.-B.); mariaantonia.martinez1@gmail.com (M.A.M.-S.); mariaa.nunez@imib.es (M.Á.N.-S.); 2 Obesity, Diabetes and Metabolism Laboratory, Biomedical Research Institute of Murcia (IMIB), 30120 Murcia, Spain; 3 Department of Digestive Diseases, Virgen de la Arrixaca University Clinical Hospital, 30120 Murcia, Spain; cristina.martinezpascual@gmail.com (C.M.-P.); juanegeavalenzuela@gmail.com (J.E.-V.); 4 Department of Biochemistry, Molecular Biology B and Immunology, Faculty of Medicine, University of Murcia, 30120 Murcia, Spain

**Keywords:** Crohn’s disease, exclusion diet, gut microbiome, corporal composition

## Abstract

**Background:** Crohn’s disease (CD) is an inflammatory bowel disease in which there is an alteration in the homeostasis and functionality of the intestinal mucosa accompanied by a dysbiosis of the commensal microbiota. The analysis of different dietary strategies to achieve CD remission and reduce gastrointestinal symptoms concludes that it is necessary to restrict the intake of ultra-processed products and to promote the consumption of those with anti-inflammatory effects that improve intestinal permeability and dysbiosis. **Methods:** Based on previous studies conducted in other cohorts, mainly pediatric, we propose an experimental, prospective, randomized study in patients with active CD who do not show improvement with conventional pharmacological treatment. The control group will receive standard nutritional recommendations while the intervention group will be prescribed an exclusion diet supplemented with enteral nutrition. **Results:** Patients in the intervention group are expected to exhibit increased lean body mass and reduced visceral fat, as measured by bioelectrical impedance analysis (BIA), alongside higher rates of clinical remission (CDAI), decreased inflammatory markers, and improved gut microbiota composition. Additionally, improvements in health-related quality of life are anticipated, as assessed by validated questionnaires. **Conclusions:** In the present project, we plan to conduct a detailed study to determine the potential of the exclusion diet for the treatment and remission of CD in adult patients, with the hypothesis that this nutritional intervention will be able to modify and improve intestinal dysbiosis, inflammatory status, and clinical and body composition markers in these patients.

## 1. Introduction

Crohn’s disease (CD) is a chronic, relapsing-remitting inflammatory disorder characterized by intestinal lesions that may appear discontinuously anywhere along the gastrointestinal tract, potentially causing chronic abdominal pain, diarrhea, intestinal obstruction, and/or perianal lesions [1].

Its multifactorial etiology results from the interaction of genetic factors, the immune system, and the individual’s commensal microbiome [2]. In turn, these are influenced by environmental factors (diet, pollution, lifestyle habits), which give rise to an aberrant response and the consequent intestinal inflammation characteristic of the disease [2].

Therefore, the development of diets aimed at modifying the gut microbiome has emerged as a promising strategy to improve the management and evolution of CD, as most drug therapies are associated with significant health costs and side effects due to their immunosuppressive role that may affect the overall health of the patient [3]. These factors, coupled with the fact that there is a considerable rate of CD patients who do not show an adequate therapeutic response to monoclonal antibodies, make absolutely necessary the development of new, less aggressive but more effective strategies to improve the quality of life of CD patients [3].

The analysis of various dietary strategies addressed to achieve remission of CD and reduce gastrointestinal symptoms concludes that it is necessary to restrict the intake of ultra-processed products while promoting foods with anti-inflammatory effects that improve intestinal permeability and dysbiosis [4,5]. The European Society for Clinical Nutrition and Metabolism (ESPEN) acknowledges that there is insufficient evidence to recommend a specific diet and emphasizes the importance of individualization. The current scientific literature supports the use of the exclusion diet (ED) in CD, which is characterized by the exclusion of frozen or packaged foods due to their additive content and the inclusion of fresh, fiber-rich foods, owing to the benefits observed in symptom remission in the pediatric population [6].

However, evidence in adults, although encouraging, remains limited [5]. The ED is supplemented with a specific enteral nutrition formula that should not exceed 1250 Kcal/day and is administered at a proportion of 25–50%, depending on the phase of the diet. The first two phases last 6 weeks each (12 weeks in total) and include foods that must be consumed daily. In the final maintenance phase, starting from week 13, there are no mandatory foods, and a Mediterranean diet is promoted [7]. The literature also advocates for modifying the dietary pattern by reducing ultra-processed foods and adhering to the Mediterranean diet after one year of initiating the ED [7,8].

On the other hand, evidence regarding the impact on body composition in patients with CD is scarce and heterogeneous, which justifies further research and the publication of higher-quality data [9,10]. These findings could present an opportunity to improve the treatment of patients with CD and to incorporate body composition assessment into routine clinical practice.

In the present clinical trial, we propose to conduct a detailed study to determine the potential of the exclusion diet for the treatment and remission of CD in adult patients, with the hypothesis that this nutritional intervention will be capable of modifying and improving intestinal dysbiosis, the inflammatory state, and both clinical and body composition markers in these patients. By assessing the impact of the exclusion diet not only on clinical remission and inflammation, but also on body composition, this study addresses multiple unmet clinical needs: it tests a low-risk, non-invasive therapeutic strategy; explores its relationship with the microbiota; and introduces a precision nutrition approach to the treatment of CD. Together, these aspects position this study as a significant contribution to the advancement of personalized medicine in CD.

## 2. Materials and Methods

### 2.1. Study Objectives

The primary objective of this clinical study is to determine the effectiveness and impact on body composition of implementing an ED for symptomatic remission in adult patients with active CD.

To achieve this objective, five specific aims are defined:

1. To assess the improvement of clinical and inflammatory parameters related to the progression of the disease.
2. To evaluate the incidence of sarcopenia through the assessment of muscle strength, function, and composition using functional tests, dynamometry, bioelectrical impedance analysis (BIA), and nutritional ultrasound.
3. To determine the rate of improvement in the quality of life of patients and the disease remission by evaluating the Crohn’s Disease Activity Index (using the CVEII9 quality of life questionnaire and the Harvey–Bradshaw Index).
4. To analyze the modifications in the intestinal microbiota resulting from the implementation of the ED.
5. To examine the progression of clinical, anthropometric, analytical, and body composition parameters.

### 2.2. Design

This is an experimental, prospective, randomized study with matched data, including patients with CD who are either about to begin pharmacological treatment or are scheduled to have their medication changed due to an insufficient response and lack of positive disease progression. The pharmacological treatment will be prescribed based on clinical indications by the medical team at baseline and will not be influenced by whether the exclusion diet is implemented.

### 2.3. Study Setting

Study participants will comprise adult patients of both sexes recruited by the Endocrinology and Nutrition Service at the Virgen de la Arrixaca University Clinical Hospital (HCUVA). These participants will be selected from individuals previously diagnosed with CD by the hospital Digestive System Service who satisfy the eligibility criteria of the study. Upon signing the informed consent document and after confirming that all inclusion criteria are met—with no exclusion criteria present—the patient will be deemed eligible for enrollment in the study. Subsequently, each enrolled patient will be assigned a unique, consecutively numbered identifier.

### 2.4. Eligibility Criteria

#### 2.4.1. Inclusion Criteria

The following criteria must be met for inclusion in this study:

- Subjects of both sexes over 18 years of age.
- A diagnosis of active luminal CD with small bowel involvement, with or without colonic involvement, prior to study inclusion.
- Active symptoms of CD at the time of initiation of the nutritional intervention.
- Active disease, defined as a Harvey–Bradshaw Index (HBI) > 4 and an objective measure of disease activity, such as an elevated inflammatory marker (CRP > 5 mg/L or 0.5 mg/dL, or calprotectin ≥ 250 µg/g) and/or a radiological imaging test (MR enterography or intestinal ultrasound) or an endoscopic test (ileocolonoscopy or capsule endoscopy).
- Ability and willingness to adhere to one of the nutritional interventions.
- Capacity to complete and sign the informed consent form.

#### 2.4.2. Exclusion Criteria

Patients who fulfill any of the following criteria will be excluded from this study:

- Patients experiencing a severe flare associated with fistulizing tracts or strictures during the study period.
- Hospitalized patients.
- Patients with known intolerance or hypersensitivity to the components of the nutritional supplement Modulen IBD.
- Patients following another diet or who are participating in other nutritional trials.
- Patients scheduled for surgical intervention during the study period.
- Patients with active malignancy.
- Patients under treatment with antibiotics or probiotics.
- Patients with other clinical conditions that may interfere with the implementation of the planned nutritional interventions (such as heart disease, celiac disease, uncontrolled diabetes, active infections, tuberculosis, or a positive stool test for Clostridium difficile toxin).

### 2.5. Withdrawal and Replacement of Study Subjects

Study subjects may withdraw from the study at any time, with or without providing a reason, without incurring any penalty or adverse consequences. Participants may revoke their consent at any time without the obligation to justify their decision and without any resulting liability or detriment. Subjects who withdraw from the study will not undergo further follow-up and will not be replaced. The investigator reserves the right to withdraw a subject from the study if it is determined that the subject can no longer comply with all study requirements or if any study procedures are deemed potentially harmful. Data collected up to the point of withdrawal will be retained and included in the analysis; however, no additional data will be gathered from the subject following withdrawal.

### 2.6. Withdrawal Criteria

Subjects will be withdrawn from this study if any of the following criteria are met:

- The occurrence of an adverse event which, in the judgment of the investigator, necessitates the withdrawal of the subject.
- A protocol deviation that compromises the interpretation of the study results and the scientific validity of the study.
- The voluntary decision of the subject.
- The explicit refusal of the subject to continue his/her participation in the study.
- Loss to follow-up.

### 2.7. Consent

Before any study-specific tests or procedures are performed, patients who meet the participation criteria (or their witness or legal representative) will be asked to sign the informed consent document approved by the Ethics Committee. They will be given sufficient time to review the informed consent document and have their questions answered before signing.

Each individual will be informed both orally and in writing about the study methodology, as well as about the potential adverse effects that may occur as a result of the various assessments to be carried out. Similarly, they will be informed that participation in the study is voluntary, both in terms of joining the study and of withdrawing from it at any time. In addition, all participants will be made aware of the characteristics of the products they will be consuming and the possible adverse effects that may occur during their use. Each participant will sign an informed consent form for participation in the project and an additional one for each stress test performed.

### 2.8. Research Ethics Approval

This study will be carried out in the HCUVA in accordance with the current Spanish legislation that regulates the carrying out of biomedical research projects, for which this protocol is established as a reference document for review by the Ethical Committees, as well as for the practical decision-making in the management of included patients by participating researchers.

### 2.9. Protocol Amendments

Except for those emergency situations, no protocol changes or deviations will be allowed without documented approval. The Research Ethics Committee must be informed of possible changes and will approve in writing any change or deviation that may increase the risks of the subject and/or may adversely affect the rights of the volunteer or the validity of the research. This stipulation does not apply to those changes that are made to reduce the inconvenience or avoid risks to the subjects and to changes that would affect the administrative aspects of the study. This study will be conducted respecting the rules of Good Clinical Practice (GCP) and the regulations and recommendations that appear in the Declaration of Helsinki and that are included in the current legislation on biomedical research at all times.

### 2.10. Intervention

Upon consenting to participate, patients will be randomized into two study arms based on the intervention administered:

- Control Group: Patients will receive modifications to their pharmacological treatment alongside standard nutritional recommendations. The control group will follow a Mediterranean diet with increased caloric and protein intake during the acute flare. The diet will emphasize a balanced nutritional intake without unnecessary food restrictions, along with adequate fiber and hydration (as tolerated), and a reduction in ultra-processed food consumption.
- Experimental Group: Patients will receive modifications to their pharmacological treatment and will be assigned to an intervention consisting of an exclusion diet in conjunction with supplemental enteral nutrition. This nutritional strategy will involve a progressive increase in the caloric intake derived from the diet, coupled with a corresponding reduction in supplemental enteral nutrition.

**Figure 1:**
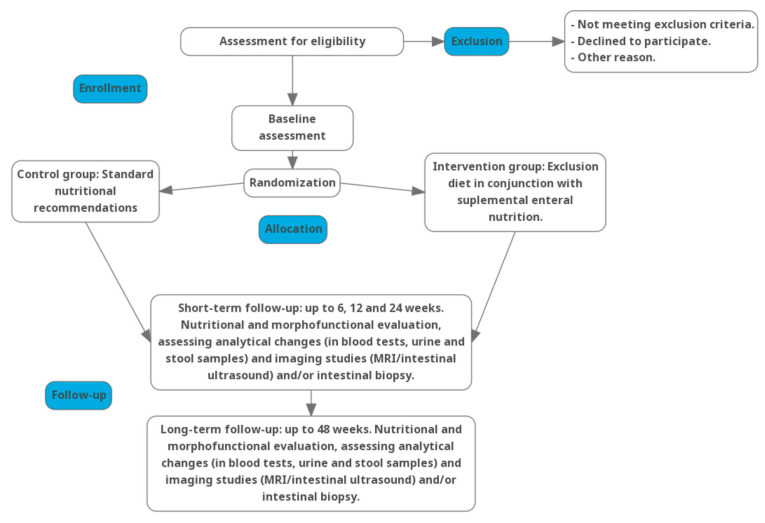
Flowchart of the trial intervention.

Prior to initiating the nutritional intervention—and subsequently at 6 and 12 weeks after the onset of the diet—alterations in body composition and functionality will be evaluated through anthropometric measurements and biochemical parameters pertinent to both the underlying pathology and the overall health status of the patient. Comprehensive functional assessments, including dynamometry and additional analyses such as BIA and nutritional ultrasound, will be conducted. Furthermore, a detailed nutritional and morphofunctional evaluation will be performed 24 and 48 weeks after the initiation of the exclusion diet [Figure 1].

Baseline biological samples (blood, urine, and stool specimens) will be collected before the start of the nutritional intervention. During the intervention, additional collections will occur at the 6-, 12-, 24-week visits, with a final sample collection at 48 weeks. A detailed assessment schedule can be found in Table 1.

### 2.11. Sample Size and Recruitment

Based on similar studies conducted primarily in pediatric populations, but also in adults [11,12], and taking into account the multiple outcomes to be obtained from the analysis of multiple variables, we estimate that, to achieve a significance level of 0.05 and a statistical power (β) of 0.8, a sample size of 48 patients per group would be required. Adjusting for a 5% dropout rate, we plan to recruit at least 50–55 patients per group to ensure the robustness of the analysis.

### 2.12. Randomization and Blinding

Patients will be randomized in a 1:1 ratio after meeting the study inclusion and exclusion criteria. In the control group, patients will receive standard nutritional recommendations, whereas in the intervention group, they will follow a dietary strategy based on partial enteral nutrition combined with an exclusion diet, which has been shown to be effective in inducing remission, promoting mucosal healing, and serving as a rescue therapy for patients with inadequate response to biologic treatments.

Patients in the intervention group will be required to maintain this dietary strategy for 12 weeks, with structured follow-up visits at 6, 12, 24, and 48 weeks. Subsequently, patients should continue with the maintenance phase starting from week 13 onwards. Nutritional intake will be adjusted to ensure a balanced provision of macronutrients and essential micronutrients, prioritizing the inclusion of key substrates for short-chain fatty acid production while avoiding foods and additives with potential pro-inflammatory effects.

Simple randomization will be computer-generated and conducted by a member of the research team at the study site. Due to the nature of the intervention, neither patients nor research or medical staff will be blinded. Group assignment will be disclosed to patients at the moment of their participation acceptance.

Furthermore, to minimize the risk of bias, the clinicians from the Gastroenterology Department responsible for performing imaging assessments, as well as the translational researchers from the Biomedical Research Institute of Murcia (IMIB) in charge of analyzing blood, urine, and stool samples, will remain blinded to the dietary allocation of the study participants.

### 2.13. Data and Sample Collection and Analysis of Variables

#### 2.13.1. Data Collection

**Figure 2:**
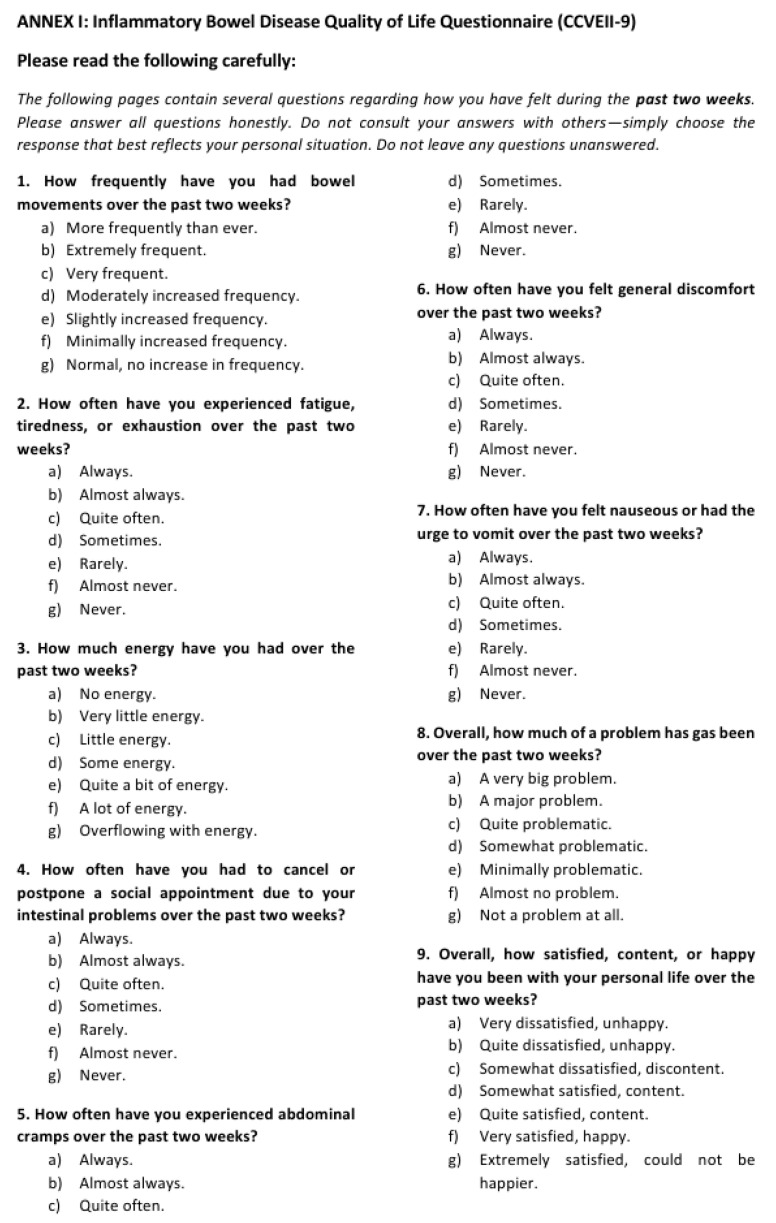
Questionnaire of quality of life (CVEII-9).

**Figure 3:**
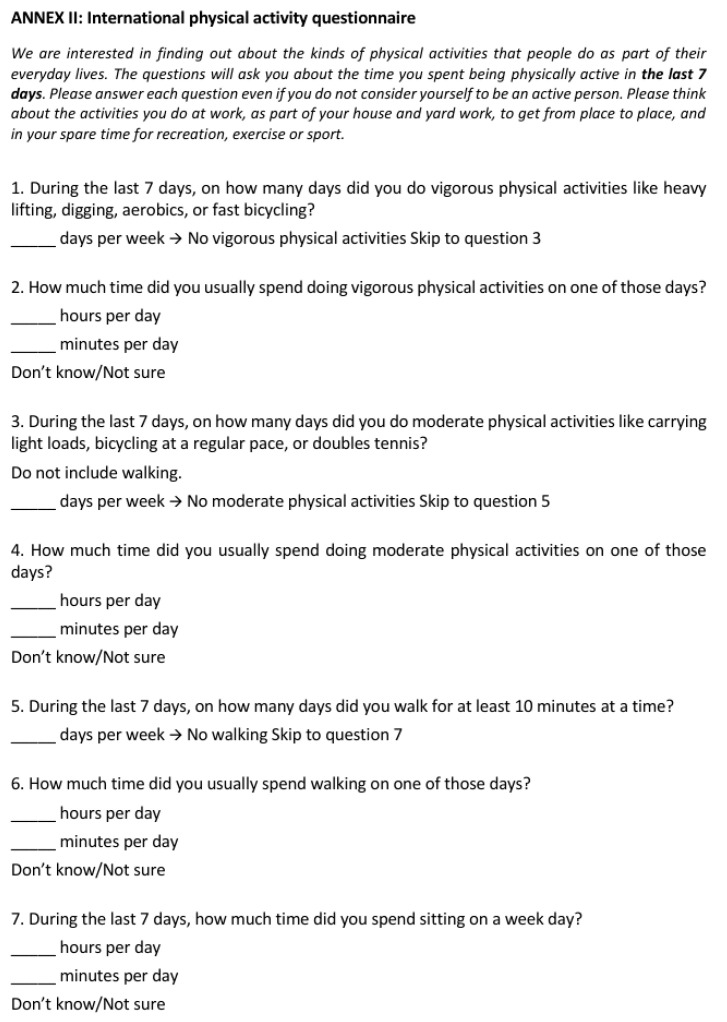
International physical activity questionnaire (IPAQ).

Data and biological sample collection will be conducted prior to the initiation of the nutritional intervention (baseline) and throughout the intervention during scheduled follow-up visits at 6, 12, 24, and 48 weeks. A comprehensive clinical assessment will be performed, encompassing the collection of anthropometric data (height, weight, body mass index), detailed records of patient medication, and evaluation of dietary intake using a 24 h dietary recall and Food Frequency Questionnaires (FFQs). Additionally, validated instruments will be employed to assess quality of life (CVEII-9) [Figure 2] and physical activity levels (IPAQ) [Figure 3].

**Figure 4:**
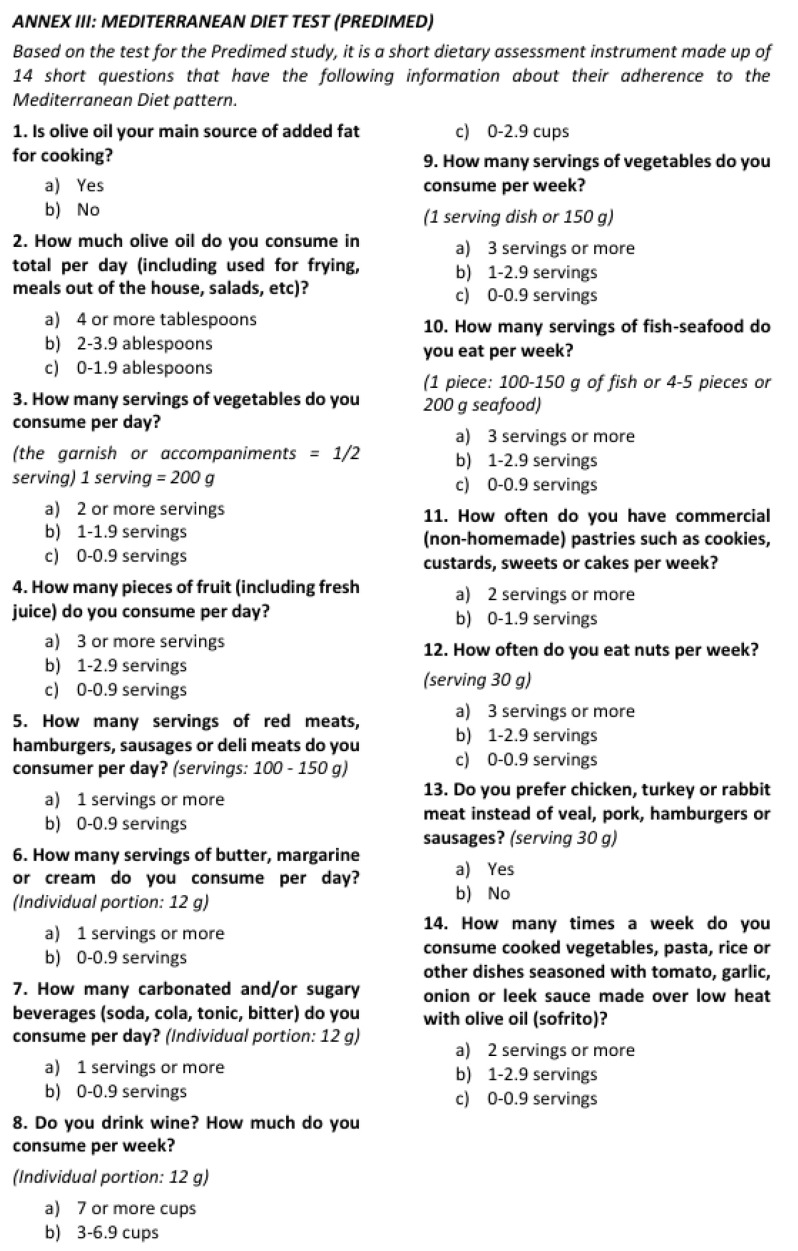
Mediterranean diet adherence questionnaire (PREDIMED).

The use of Mediterranean diet adherence questionnaires (PREDIMED) [Figure 4], the 24 h dietary record, and Food Frequency Questionnaires (FFQs) are useful tools for assessing adherence to the Mediterranean diet. However, they are not without limitations, as they may not capture inter-individual variability in diet or may be misinterpreted by subjects. Measurement of certain biomarkers that increase during a Mediterranean diet (such as carotenoids, n-3 fatty acids, and/or monounsaturated fatty acids) may be a more objective alternative to assess dietary adherence [13].

Body composition and functional changes will be analyzed using biochemical parameters associated with the underlying pathology and the patient’s general health status. Functional performance tests, including dynamometry, BIA, and nutritional ultrasonography, will be conducted to evaluate morphofunctional alterations. A comprehensive nutritional and morphofunctional assessment will be repeated three months following the conclusion of the dietary intervention.

Clinical remission will be evaluated using the Harvey–Bradshaw Activity Index (score < 5) and fecal calprotectin levels (<250 µg/g). At weeks 24 and 48, the radiological imaging study performed at baseline (e.g., MRI enterography or intestinal ultrasonography) will be repeated and/or an intestinal biopsy may be performed to objectively confirm remission of CD.

#### 2.13.2. Sample Collection and Storage

Biological samples, including blood, urine, and stool, will be collected at baseline (prior to the initiation of the nutritional intervention) and at scheduled follow-up visits at 6, 12, 24, and 48 weeks. Sample collection will adhere to standardized protocols to ensure the integrity and reproducibility of the analyses.

Blood samples will be collected via venipuncture into EDTA and serum separator tubes. Plasma and serum will be separated by centrifugation at 4 °C within 30 min of collection. Stool samples will be obtained using sterile containers and immediately aliquoted for microbiota, calprotectin, and metabolomic analyses. Urine samples will be collected using sterile procedures and processed within 1 h of collection. All biological materials will be aliquoted into cryovials and stored at −80 °C until further analysis.

Sample transportation from the collection site to the laboratory will be conducted using validated cold-chain logistics to maintain a temperature of 2–8 °C for short-term handling and ensure sample stability. Metadata associated with each sample (e.g., time of collection, participant ID, processing time) will be systematically recorded in a digital database for traceability.

To ensure long-term preservation, aliquots will be stored in a dedicated biobank facility under controlled conditions with continuous temperature monitoring and backup power systems. Samples will be retained for subsequent analyses, including but not limited to biochemical assays, genomic and proteomic studies, and microbiota profiling. All procedures comply with international ethical guidelines and Good Laboratory Practices (GLPs).

#### 2.13.3. Characterization of Quantifiable Analytical Parameters in Serum, Urine, and Stool

The following hematological, biochemical, metabolic, nutritional, and inflammatory parameters will be quantified in serum:

- Hematological and coagulation parameters: complete blood count (CBC) and coagulation profile.
- General biochemical profile: fasting glucose, electrolytes (sodium, potassium, chloride), nitrogenous compounds (urea, creatinine), mineral metabolism markers (calcium, phosphorus, magnesium), hepatic function enzymes (aspartate aminotransferase [AST/GOT], alanine aminotransferase [ALT/GPT], gamma-glutamyl transferase [GGT], alkaline phosphatase, total and direct bilirubin, lactate dehydrogenase [LDH], creatine kinase).
- Metabolic and endocrine markers: insulin, homeostatic model assessment for insulin resistance (HOMA-IR), C-peptide, and glycated hemoglobin (HbA1c).
- Lipid profile: total cholesterol, high-density lipoprotein cholesterol (HDL-C), low-density lipoprotein cholesterol (LDL-C), and triglycerides.
- Nutritional biomarkers: prealbumin, albumin, fat-soluble vitamins (A, D, E), trace elements (zinc, selenium), vitamin B12, iron, folate, ferritin, transferrin, and transferrin saturation index.
- Inflammatory biomarkers: C-reactive protein (CRP), erythrocyte sedimentation rate (ESR), and interleukin-6 (IL-6). Inflammatory markers will be quantified using high-sensitivity multiplex immunoassays to enhance precision and reduce inter-assay variability.

Analysis in fecal samples will include the following:

- Fecal calprotectin: a surrogate marker of intestinal inflammation, measured using enzyme-linked immunosorbent assay (ELISA) or equivalent high-sensitivity immunoassays.
- Analysis of the gut microbiota composition will be performed by 16S rRNA gene sequencing in stools. DNA extraction from stool samples will be performed using 200 mg of sample using the QIAamp DNA Stool Mini kit (Qiagen, Manchester, UK), following the manufacturer’s instructions. The concentration and quality of the DNA will be determined by the Agilent 2200 TapeStation system. To create the libraries, DNA samples (5 ng/µL) will be amplified using a primer, encoding the hypervariable regions V2-4-8 and V3-6, 7-9 of the bacterial 16S rRNA using the 16S Metagenomics Kit (Thermo Fisher, Waltham, MA, USA). The amplicons obtained will be purified using AMPure^®^ XP beads (Beckman Coulter, Pasadena, CA, USA) and, subsequently, the libraries will be created using the Ion Plus Fragment Library kit (Thermo Fisher, Waltham, MA, USA) and the Ion Xpress Barcode Adapters 1–16 kit (Thermo Fisher, Waltham, MA, USA) to add barcodes to purified amplicons. The libraries will be purified using AMPure^®^ XP beads (Beckman Coulter, Pasadena, CA, USA) and quantified by fluorescence. Sequencing of the libraries will be carried out using an Ion S5 platform. The results of composition, diversity, and richness will be analyzed using the QIIME2 platform, and functionality will be analyzed through the PICRUST software package (Version v2.6.2).

### 2.14. Data Management

An electronic Case Report Form (eCRF) will be used to systematically record all study-related variables. A structured data collection form will be designed and implemented in SPSS software for statistical processing. Upon completion of this study, all collected data will be reviewed and analyzed for inclusion in the final study report.

All study-related documents will be securely stored in the Principal Investigators Archive at the participating institution, under the custody of the Principal Investigators, until this study is completed. Once the study concludes, the documentation will be indexed and transferred to the central archive of the institution, in compliance with Good Clinical Practice (GCP) guidelines.

Data will be iteratively verified and corrected until the database is fully validated. Following data cleaning and validation, variables will be recoded and transformed (e.g., grouping, summation, composite indices) for further statistical analyses.

### 2.15. Statistical Methods

All statistical analyses will be performed using SPSS software version 22.0, considering *p* < 0.05 as the threshold for statistical significance. A descriptive analysis of the variables will be conducted, providing point estimates and 95% confidence intervals. Continuous variables will be expressed as means and standard deviations or medians depending on the distribution of the data. Categorical variables will be reported as frequencies and percentages.

Comparisons of continuous variables will be performed using Student’s *t*-test (for two-group comparisons) or ANOVA (for comparisons involving more than two groups). Associations between categorical variables will be assessed using the chi-square test. Correlation analyses will be conducted using Pearson’s or Spearman’s correlation coefficients, depending on the normality of the variables under study. Relationships between multiple variables will be analyzed using multivariate statistical models.

Given the multiple outcomes to be assessed and the multiple comparisons to be analyzed, a Bonferroni correction (0.05/no. of comparisons as the significance level) will be also performed to avoid the risk of false positives.

## 3. Discussion

The findings of this study could contribute to the implementation of nutritional therapy in adult patients with active luminal CD (non-stricturing, non-penetrating) of the small intestine, with or without colonic involvement, who are initiating treatment with biologic agents or JAK inhibitors. Partial enteral nutrition (PEN) combined with the exclusion diet (ED) has demonstrated efficacy in inducing remission in both pediatric and adult patients, promoting mucosal healing and serving as a rescue therapy for patients who fail biologic therapy [11,12].

In this context, the Crohn’s Disease Exclusion Diet (CDED) has emerged as a promising whole-food-based dietary intervention, specifically designed to reduce exposure to dietary components that impair the intestinal barrier and alter the gut microbiome. In a pivotal 12-week prospective randomized trial, Levine et al. compared CDED combined with partial enteral nutrition (PEN) to exclusive enteral nutrition (EEN) in children with mild to moderate CD [12]. The study showed that CDED + PEN was significantly better tolerated than EEN (97.5% vs. 73.6%, *p* = 0.002), and while both approaches achieved comparable remission rates at week 6, sustained corticosteroid-free remission at week 12 was notably higher in the CDED + PEN group (75.6% vs. 45.1%, *p* = 0.01) [14]. Additionally, the CDED + PEN group exhibited sustained reductions in inflammatory biomarkers and favorable modulation of the fecal microbiota [12]. These findings support the clinical utility of CDED + PEN as a more tolerable and effective strategy for inducing and maintaining remission in pediatric CD. Recent clinical data on adult patients reinforce the efficacy of CDED beyond pediatric populations. In a study involving 32 participants (18 women and 14 men), clinical remission was achieved in 76.7% of patients at 6 weeks and in 82.1% at 12 weeks of therapy [11]. Furthermore, fecal calprotectin levels showed a significant reduction by the second follow-up time point compared with baseline (*p* = 0.021) [11]. These findings suggest that CDED is also an effective therapeutic option for inducing remission in adult patients with CD.

The primary advantage of this dietary strategy lies in its balanced, sustainable, and palatable nature, making it easier to adhere to over time. This is largely due to its inclusion of dietary fiber and essential substrates necessary for the production of short-chain fatty acids [10]. The exclusion diet is based on the elimination or inclusion of specific dietary components while ensuring a nutrient composition that supports the growth and maintenance of lean body mass [15,16].

Foods and additives that should be excluded from this diet include those associated with high fat intake (particularly from animal sources, such as red meat), dairy products, wheat, alcohol, yeast, and insoluble fiber [16]. Additionally, food additives recommended for avoidance include emulsifiers, carrageenan, maltodextrins, sulfites, and titanium dioxide [16]. Conversely, the diet should be low in taurine, rich in proteins and complex carbohydrates, and free of gluten or modified starches [15]. Thus, the CDED consists of a series of obligatory, permitted, and prohibited foods that can be eaten at different phases of the diet as detailed in Table 2.

On the other hand, the available evidence regarding the impact of CD on body composition remains scarce and heterogeneous, highlighting the need for further high-quality research and data publications [12,13,17,18]. These findings could represent an opportunity to enhance the management of CD and integrate body composi-tion assessment into routine clinical practice. The effect of the exclusion diet on muscle quantity, quality, and functionality may be related to the patient’s quality of life, and its monitoring and follow-up is a guiding parameter in nutritional intervention by in-creasing the intake of certain macronutrients (such as protein). Information on body composition would allow the personalization of nutritional intervention.

This study presents some methodological limitations. First, the use of self-administered dietary assessment tools (PREDIMED and FFQ) introduces inherent biases, including recall bias and social desirability bias, which may compromise the accuracy of reported dietary adherence. Second, inter-individual variability in adherence to the nutritional intervention could affect the interpretation of objective outcome measures. Additionally, attrition and a relatively limited sample size may reduce the statistical power of the study, potentially limiting the ability to detect clinically meaningful differences or perform robust subgroup analyses. Residual confounding variables—such as psychological stress, levels of physical activity, and other lifestyle factors—may also influence study outcomes despite attempts at control. Finally, both inter- and intra-observer variability in ultrasound assessments, including intestinal ultrasound and quadriceps rectus femoris muscle evaluation, constitute further sources of potential measurement bias.

## Figures and Tables

**Table 1:** Proposal for the follow-up of adult Crohn’s disease patients included in this study.

Study Period						
Timepoint	Pre-DE	t0	t1 (6 w)	t2 (12 w)	t3 (24 w)	t4 (48 w)
**Enrollment**						
Diagnosis (Index, Harvey–Bradshaw Index, MUST)	X					
Inclusion criteria and exclusion criteria	X					
Informed consent signature	X					
**Intervention**						
Standard nutritional recommendations vs. ED + enteral nutrition		X	X	X	X	X
**Assessments**						
Quality of life questionnaire (CVEII-9)		X	X	X	X	X
Harvey–Bradshaw activity index		X	X	X	X	X
Physical activity questionnaire (PAQ)		X			X	X
Food Frequency Questionnaire (FFQ)		X			X	X
24 h dietary recall		X	X	X	X	X
Mediterranean diet adherence questionnaire (PREDIMED)			X		X	X
Anthropometry (weight, height, BMI)		X		X	X	X
Sarcopenia screening (SARC-F)		X		X	X	X
Functional sarcopenia tests (dynamometry)		X		X	X	X
Bioelectrical Impedance Analysis (BIA)		X	X	X	X	X
Nutritional ultrasound		X		X	X	X
Blood tests, urine and stool samples		X	X	X	X	X
Intestinal imaging (MRI/intestinal ultrasound).		X			X	X
Intestinal biopsy. Optional.		X			X	X

Pre-DE: before diet exclusion. t0: baseline or initial visit. t1 (6 w): visit at 6 weeks. t2 (12 w): visit at 6 weeks. t3 (24 w): visit at 24 weeks. t4 (48 w): visit at 48 weeks.

**Table 2:** Obligatory, permitted, and prohibited foods in the exclusion diet in patients with Crohn’s disease.

Phase	Type of Food	
Phase 1 (0–6 weeks)	Obligatory	Protein-rich foods: fresh chicken breast (at least 150–200 g/day), 2 eggs/day. Carbohydrate-rich foods: 2 fresh potatoes/day peeled, cooked and cooled before consumption, 2 bananas/day, 1 apple/day peeled.
	Permitted	Protein-rich foods: 100–150 g of fresh white fish once a week as a substitute for chicken. Carbohydrate-rich foods: white rice (unlimited), rice noodles without preservatives (unlimited), rice flour for baking (unlimited). Fruits: 1 avocado/day (no more 1/2 avocado per meal), 5 ripe strawberries/day, 1 cantaloupe slice/day. Vegetables: 2 tomatoes/day (or 6 cherry tomatoes), 2 cucumbers/day peeled, 1 carrot/day, fresh spinach (225 g raw leaves/day), 3 lettuce leaves once a day. Condiments: olive oil, canola oil, salt, pepper, paprika, cinnamon, cumin, turmeric, mint, oregano, coriander, rosemary, sage, basil, thyme, dill, parsley, onion (all types), garlic, ginger, natural lemon juice, honey (3 tbsp/day), sugar (3 teaspoons/day). Drinks: water, sparkling water, infusions, 1 glass of freshly squeezed orange juice per day.
	Prohibited	Protein-rich foods: pre-cooked or smoked processed meat and fish, seafood, red meat, pork, pork, turkey, and other poultry parts, soy products, dairy products, ice cream, vegetable milks (soy, rice, almond). Carbohydrate-rich foods: wheat products (breakfast cereals, bread and baked goods of any kind), baker’s yeast and other flours, gluten-free products not mentioned above, soya products, pulses (lentils, peas, chickpeas and beans), maize, frozen potatoes. Fruits: dried fruits, all other fruits. Vegetables: frozen vegetables, kale, leek, asparagus, artichokes, all other vegetables not mentioned as permitted. Condiments: margarine, sauces, salad dressings, syrups (maple syrup, corn syrup, etc.), jam of any kind, artificial sweeteners, spice mixtures, other oils (soybean oil, sunflower oil, corn oil, etc.), marmalade of any kind, artificial sweeteners and oil spray. Beverages: soft drinks, fruit juice, other sweetened beverages, alcoholic beverages, coffee, chocolate milk, milkshakes, all types of tea (leaf and tea bags). Nuts and dried fruit. Others: canned goods, packaged snacks (crisps, crackers, popcorn, etc.), candy, chocolate, cakes, biscuits, chewing gum. Foods that are not on the list of recommended or permitted foods are prohibited.
Phase 2 (7–12 weeks)	Obligatory	Same as phase 1.
	Permitted	Protein-rich foods: 100–150 g of fresh white fish once a week as a substitute for chicken, one can of tuna in olive or canola oil once a week. Eating red meat is not recommended, but if you wish, you should limit yourself to a fresh fillet of unprocessed lean meat, up to 200 g per week (only once a week). Carbohydrate-rich foods: white rice (unlimited), rice noodles without preservatives (unlimited), rice flour for baking (unlimited), ½ sweet potato/day, bread 1 slice/day (preferably homemade), lentils, peas, chickpeas or dried beans, half a cup/day uncooked, quinoa (unlimited), 50 g oats (one serving of oatmeal or crackers is allowed 1 or 2 times a day). Fruits: 1 avocado/day (no more 1/2 avocado per meal), 5 ripe strawberries/day, 1 cantaloupe slice/day, one pear, peach or kiwi fruit per day, 10 blueberries (25 g) can replace the allowed strawberries. From week 10 onwards, all fruits can be introduced in small quantities (except those on the off-limits list). Vegetables: 2 tomatoes/day (or 6 cherry tomatoes), 2 cucumbers/day peeled, 1 carrot/day, fresh spinach (225 g raw leaves/day), 3 lettuce leaves once a day, courgette (1 large or 2 small), 4–6 fresh mushrooms, 2 broccoli or cauliflower florets (but not all at the same time). From week 10 onwards, all vegetables can be introduced in small quantities (except those on the off-limits list). Condiments: olive oil, canola oil, salt, pepper, paprika, cinnamon, cumin, turmeric, mint, oregano, coriander, rosemary, sage, basil, thyme, dill, parsley, onion (all types), garlic, ginger, natural lemon juice, honey (3 tbsp/day), sugar (3 teaspoons/day), baking soda or baking powder. Drinks: water, sparkling water, infusions, 1 glass of freshly squeezed orange juice per day, chamomile tea (permitted from week 7 onwards). Nuts: unsalted, unroasted, and unprocessed almonds or walnuts, 36 g/day Tahini (without emulsifiers and sulfites).
	Prohibited	Protein-rich foods: pre-cooked or smoked processed meat and fish, seafood, red meat, pork, pork, turkey, and other poultry parts, soy products, dairy products, ice cream, vegetable milks (soy, rice, almond). Carbohydrate-rich foods: wheat products (breakfast cereals, bread and baked goods of any kind), baker’s yeast and other flours, gluten-free products not mentioned above, soya products, pulses (lentils, peas, chickpeas and beans), maize ((permitted from week 10), frozen potatoes. Fruits: dried fruits, all other fruits (permitted from week 10 onwards). Fruit is not permitted under any circumstances: persimmon, passion fruit, pomegranate, prickly pear. Vegetables: frozen vegetables, kale, leek, asparagus, artichokes. Condiments: margarine, sauces, salad dressings, syrups (maple syrup, corn syrup, etc.), jam of any kind, artificial sweeteners, spice mixtures, other oils (soybean oil, sunflower oil, corn oil, etc.), marmalade of any kind, artificial sweeteners and oil spray. Beverages: soft drinks, fruit juice, other sweetened beverages, alcoholic beverages, coffee, chocolate milk, milkshakes, all types of tea (leaf and tea bags). Nuts and dried fruit. Others: canned goods, packaged snacks (crisps, crackers, popcorn, etc.), candy, chocolate, cakes, biscuits, chewing gum. Foods that are not on the list of recommended or permitted foods are prohibited.
Phase 3 (13 week)	Permitted	The phase 2 diet will be followed for 5 days of the week (Monday to Friday) with the following additions: Protein-rich foods: other parts of the chicken can be used, but the skin, wings, and offal should be avoided; fresh seafood, white fish or salmon, once a week, one serving per day of natural unprocessed yogurt with all-natural fat and no additives. Carbohydrate-rich foods: two slices of bread per day (preferably homemade), a small portion of pasta can be eaten instead of bread (200 g cooked). Fruits: all fruits and berries, including dehydrated fruits if they are sulfite-free, except for fruits that are expressly not allowed. Vegetables: all vegetables except those expressly not allowed (provided you do not suffer from stenosis). Drinks: one cup of black coffee or tea (no instant coffee or capsules and only 1 cup per day). Same restrictions for other foods, beverages, and condiments as in phase 2. Weekends (Saturdays and Sundays): foods that are not included in the ED are allowed at free meals. It is important not to overeat foods that are not included in the ED. Try to maintain a varied diet also during free meals at weekends. Homemade food is preferable to frozen or processed food. One free home-cooked breakfast and one free home-cooked meal (lunch or dinner) per day on Saturdays and Sundays (including wheat, dairy, fish and other types of meat).
	Prohibited	Protein-rich foods: frozen doughs (cake or pizza bases), processed meats (such as hot dogs, sausages, bacon), ready meals (frozen from fresh produce), and soft drinks. Fruits: passion fruit, persimmon, prickly pear, pomegranate. Vegetables: raw celery, large quantities of kale, leeks.

## Data Availability

Not applicable.
